# Implementation and evaluation of a mindfulness based program for children in a school setting in france

**DOI:** 10.1192/j.eurpsy.2021.1237

**Published:** 2021-08-13

**Authors:** J. Monsillion, R. Zebdi, L. Romo

**Affiliations:** Ea 4430 Clipsyd, Department Of Psychology, Université Paris Nanterre, Nanterre, France

**Keywords:** Children, Mindfulness based school interventions, emotion regulation, mental health

## Abstract

**Introduction:**

Mindfulness-based interventions (MBIs) held within an academic context continue to develop at an international scale, and continue to show benefits for children, specifically on cognitive functioning and emotional regulation (Theurel, Gimbert, Gentaz, 2018).

**Objectives:**

The aim of this study is to demonstrate the relevance of implementing MBIs within French elementary school settings, in order to promote mental health, positive pedagogy and quality of life.

**Methods:**

This study utilizes the Belgian program “Gestion des émotions par la pratique de la Pleine Conscience” (Emotional regulation using Mindfulness practices) (Deplus, 2015) and compares an experimental group participating in 9 MBI sessions to a control group “waiting list”. An interpretative phenomenological analysis at post-test investigates participant’s perception on the effects of the intervention on familial and school climates.

**Results:**

The results of this study have been highly impacted by COVID19 (confinement).Quantitative results reveal that 33% of the participants (n=6) show a decrease in depressive symptoms, 33% show no change and 33% show a slight increase, post-intervention (T2). Results also suggest a decrease in anhedonia in 50% of the participants. 50% of participants show an improvement with regards to worrying and hypersensitivity. Qualitative analysis puts forth perceived improvements on emotional regulation and mindfulness abilities of participants, which has shown to have positive effects on familial climate.

**Conclusions:**

We estimate that the implementation of such a MBI in a school setting will favour the development of executive functions and emotional regulation, allowing children to develop resilience towards stress and anxious-depressive affects. We also expect the intervention to help develop socio-emotional abilities and well-being in French schools.

